# Neoantigen-Driven Immunotherapy in Triple-Negative Breast Cancer: Emerging Strategies and Clinical Potential

**DOI:** 10.3390/biomedicines13092213

**Published:** 2025-09-09

**Authors:** Peter A. Shatalov, Anna A. Bukaeva, Egor M. Veselovsky, Alexey A. Traspov, Daria V. Bagdasarova, Irina A. Leukhina, Anna P. Shinkarkina, Maria P. Raygorodskaya, Alena V. Murzaeva, Yulia A. Mechenici, Maria A. Revkova, Andrey D. Kaprin, Peter V. Shegai

**Affiliations:** 1 National Medical Research Radiological Centre of the Ministry of Health of the Russian Federation, 249036 Obninsk, Russia; annbukaeva@gmail.com (A.A.B.); egor.veselovsky@gmail.com (E.M.V.); traspovalex@gmail.com (A.A.T.); dasha.bagdasarova@gmail.com (D.V.B.); ileuhina@gmail.com (I.A.L.); ashinkarkina@mail.ru (A.P.S.); maria.raygorodskaya@gmail.com (M.P.R.); lewnmur@gmail.com (A.V.M.); doroshenkowork227@gmail.com (Y.A.M.); marirevkova@gmail.com (M.A.R.); kaprin@mail.ru (A.D.K.); dr.shegai@mail.ru (P.V.S.); 2 Department of Urology and Operative Nephrology, Peoples Friendship University of Russia (RUDN University), 117198 Moscow, Russia

**Keywords:** triple-negative breast cancer, neoantigens, cancer immunotherapy, cancer vaccines, adoptive cell therapy, peptide vaccine, mRNA vaccine, peptide vaccine, combination therapy

## Abstract

Triple-negative breast cancer (TNBC) is one of the most aggressive subtypes of breast cancer (BC), comprising approximately 20% of newly diagnosed BC cases. The poor prognosis, high recurrence rates, and inefficacy of hormone-based therapies make TNBC one of the greatest challenges in contemporary oncology. The unique immunological features of TNBC, including relatively high tumor mutational burden, abundance of tumor-infiltrating lymphocytes, and elevated PD-L1 expression, offer a wide range of opportunities for immunotherapeutic approaches, of which the most progressive and promising are neoantigen-driven ones. This review examines the current landscape of neoantigen-based therapeutic approaches in TNBC treatment, spanning from discovery methodologies to clinical applications. We provide a critical analysis of the tumor microenvironment (TME) in TNBC, highlighting the balance between its immunoactivating (CD8+ T-cells, dendritic cells) and immunosuppressive (regulatory T-cells, M2 macrophages) components as the key determinant of therapeutic success, as well as reviewing the emerging approaches to TME reprogramming and recruiting in favor of better outcomes. We also present state-of the-art methods in neoantigen identification and prioritization, covering the landscape of technological platforms and prediction algorithms, addressing the existing accuracy limitations along with emerging computational solutions, and comprehensively discussing the TNBC neoantigen spectrum. Our analysis shows the strong domination of patient-specific (“private”) neoantigens over shared variants in the TNBC, with *TP53* as the only gene with recurrent variants. Finally, we extensively cover neoantigen-recruiting therapeutic modalities including adoptive cell therapies, personalized vaccine platforms (peptide-based, mRNA/DNA vaccines, dendritic cell vaccines), and oncolytic viruses-based approaches. Our study of current clinical trials demonstrates the substantial gap between early proof-of-concept experiments and further applicability of neoantigen-driven therapies. The major challenges hampering the success of such methods include neoantigen prediction inaccuracy rates, high manufacturing costs, and time consumption. Promising ways to overcome these difficulties include the development of combinational strategies, TME modeling and modifying, and improvement of the therapy delivery properties, along with the optimization of production workflows and cost-effectiveness of vaccine development.

## 1. Introduction

Triple-negative breast cancer (TNBC) is one of the most aggressive subtypes of breast cancer (BC), lacking the expression of estrogen receptor (ER), progesterone receptor, and human epidermal growth factor receptor 2 (HER2). Accounting for up to 20% of newly diagnosed BC cases [1], TNBC is characterized by rapid metastasis [2], no less than a 25% recurrence rate [3], poorer prognosis, and shorter survival times compared to other BCs [4,5]. Because of its receptor-negative nature, TNBC is insensitive to hormone therapy, and, thus, cytotoxic chemotherapy remains the basis of treatment [6,7]. However, the benefits of chemotherapeutic treatment may be outweighed by its low efficacy and systemic toxicities [8,9]; thus, the need for alternative, improved therapeutic approaches is essential.

In recent years, a considerable body of information has accumulated on the causes of TNBC aggressiveness: high levels of lymphocyte infiltration and frequent somatic mutations together comprise the “immunological portrait” of TNBC [10]. Recent advances in immunotherapy have offered new opportunities for targeting these specific features. In particular, the higher expression of PD-L1 in TNBC [11] provided additional grounds for therapeutic approaches based on immune checkpoint inhibitors (ICIs). Pembrolizumab was approved by the FDA after showing promising efficacy and safety in TNBC both as monotherapy [12] and combined with chemotherapeutic agents [13]. Nevertheless, despite this success, durable clinical responses to pembrolizumab in TNBC remain low (objective response rate (ORR) = 18.5% as monotherapy [14] and up to 40% in combination with chemotherapy [13]), and both innate and acquired resistance to ICIs occur [15,16]. Immunological treatment of ICIs-resistant (so-called “cold” [17]) tumors presents a major challenge in TNBC management. In recent years, efforts have been made to exploit TNBC unique immunobiology—including its relatively high tumor mutational burden (TMB) [18], PD-L1 expression [11,19], and abundance of tumor-infiltrating lymphocytes (TILs) [20,21]—to develop strategies that convert “cold” tumors into “hot,” actionable immunotherapeutic targets.

Central to this endeavor are neoantigens, or tumor-specific antigens (TSAs), which arise from somatic mutations within the tumor and enable the T-cell immune response to malignant cells. Unlike conventional tumor-associated antigens (TAAs), neoantigens do not occur in normal tissues [22,23] and neoantigen-specific T-cells elicit more effective tumor-specific immune response than TAA-specific T-cells [24]. Also, neoantigen-targeting approaches provide minimized off-target toxicity, which is a common hurdle in existing TAA-based immunotherapeutic strategies [25]. Genomic instability driven by homologous recombination deficiency (HRD) and *BRCA1/2* mutations [26,27] provides a rich repertoire of neoantigens, making TNBC a promising candidate for neoantigen-driven approaches [23]. However, the impact of immunosuppressive components of tumor microenvironment (TME), such as regulatory T-cells (Tregs) [28], dysfunctional dendritic cells [29], and M2 macrophages [30], may hamper immunotherapies by enhancing the immune evasion of tumor cells [31]. Understanding how to overcome these obstacles, along with the discovery and selection of highly immunogenic neoantigens, is critical for the further development of next-generation immunotherapy.

In this review, we cover the existing methods of neoantigen identification and selection, as well as new and emerging neoantigen-driven strategies for TNBC therapy, with a focus on cancer vaccines, adoptive cell therapies, and TME modulation. We also discuss complementary approaches where immunotherapies combine with other treatments, along with the persisting challenges such as immunogenicity issues, immune evasion, and cost-effectiveness of neoantigen-based methods.

## 2. Tumor Microenvironment in TNBC and Therapeutic Opportunities

The microenvironment of a malignant neoplasm is a complex of immune and non-immune cells along with extracellular matrix (ECM) and signaling molecules, including cytokines, chemokines, and other factors secreted by those cells. The characteristic features of TME in TNBC are high levels of TILs and tumor-associated macrophages (TAMs), and, consequently, the abundant expression of cytokines and growth factors [32]. Pro- and anti-tumorigenic populations comprising the TME in TNBC critically influence tumor antigen presentation and effector T-cell function [33,34,35]. Based on the composition and ratio of the TME components, TNBC can be divided into several molecular subtypes with different clinical and prognostic features [36,37].

### 2.1. CD8+ Cells: Main Effectors

Cytotoxic CD8+ T-cells are the main type of cells responsible for the destruction of malignancies through the recognition of tumor-derived peptides presented by MHC-I molecules [32,38]. CD8+ T-cells directly cause cytotoxicity via multiple mechanisms, including granzyme/perforin release, cell death induction, and secretion of cytokines, such as IFN-γ, TNF-α or TNF-β [39,40].

Granzyme/perforin pathway is the primary cytotoxic mechanism of CD8+ cells, triggering apoptotic cell death through rapid delivery of granzymes—serine proteases—into the target cells [41]. In addition, activated CD8+ cells express death receptor ligands such as Fas ligand and tumor necrosis factor (TNF)-related apoptosis-inducing ligand (TRAIL), which further potentiate apoptosis induction. Remarkably, the TRAIL-mediated mechanism of cytotoxicity has been of particular interest for oncologists due to its ability to induce cell death selectively in cancer cells [42]; however, the attempts to develop TRAIL-based cancer therapies failed to reach success [43]. The recent study of TRAIL effects in TNBC revealed that the TRAIL treatment promotes neutrophil-mediated immune suppression, which can explain the inefficacy of TRAIL-driven immunotherapeutic approaches [44].

Besides direct cytolysis, CD8+ T-cells may cause broader anti-tumor responses through cytokine production. The IFN-γ signaling enhances MHC class I antigen presentation on tumor cells, activates TAMs, and promotes additional recruitment of immune cells to the site of malignancy. In turn, TNF-α, one of the major apoptosis inducers, can directly induce tumor cell death, as well as enhance intratumoral vascular permeability to improve immune cell infiltration [41]. Recent studies showed that CD8+ T-cells can orchestrate ferroptosis, which is a non-apoptotic, iron-dependent form of programmed cell death, through IFN-γ-induced metabolic reprogramming in cancer cells [45].

The redundancy among the aforementioned mechanisms helps provide effective immune responses when individual pathways are compromised by tumor immune evasion strategies or immunosuppressive TME factors, which is particularly relevant in TNBC. Based on the above, CD8+ T-cells are critically important in TNBC prognosis and treatment: high intratumoral CD8+ T-cell density strongly correlates with improved survival, pathologic complete response (pCR) rates after neoadjuvant chemotherapy, and prolonged progression-free survival (PFS) with immune checkpoint inhibitors (ICIs) [46]. More specifically, the recent meta-analysis showed that high CD8+ T-cell infiltration levels in BC significantly correlated with better overall survival [hazard ratio (HR) = 0.70, 95% confidence interval (CI): 0.60–0.82, *p* < 0.001] and disease-free survival (HR = 0.63, 95% CI: 0.49–0.81, *p* < 0.001) [47]. Moreover, spatial analyses of TNBC tumors reveal that close proximity (<20 µm) of CD8+ T-cells to malignant cells predicts favorable therapeutic outcomes [48,49].

### 2.2. CD4+ Cells and Regulatory T-Cells

Unlike CD8+ T-cells, CD4+ helper T-cells mostly orchestrate immune responses indirectly by secreting cytokines or performing regulatory functions [50]. The cytokine-mediated mechanisms of action of CD4+ T-cells involve several distinct pathways. Upon activation, CD4+ T-cells differentiate into specialized subsets (Th1, Th2, Th17, and Tregs) that secrete specific cytokine profiles to coordinate immune responses. Th1 cells produce IFN-γ and IL-2 to promote cytotoxic T-cell responses and activate macrophages; Th2 cells secrete IL-4, IL-5, and IL-13 to support B-cell activation and antibody production; Th17 cells release IL-17 and IL-22 to mediate inflammatory responses; at last, Tregs produce immunosuppressive cytokines, including IL-10 and TGF-β, to develop and maintain immune tolerance. The cytokine production is controlled by transcription factors such as STAT proteins that respond to the local cytokine environment and coordinate with co-stimulatory signals to determine T-cell fate and function [51].

In contrast to CD8+, CD4+ T-cell infiltration shows mixed prognostic associations, as subsets like Tregs (CD4+FoxP3+) actively suppress CD8+ effector function through TGF-β, IL-10, and adenosine-mediated pathways, blunting anti-tumor immunity [52]. As is known, TNBCs with high TMB often generate abundant clonal neoantigens [53,54], which could be expected to prime robust CD8+ T-cell responses. However, these responses are frequently being stifled by immunosuppressive cell populations such as Tregs and M2-polarized macrophages.

### 2.3. Dendritic Cells: Antigen Presentation

Dendritic cells (DCs), the key antigen-presenting cells, play a pivotal role in neoantigen recognition and positively correlate with TIL levels and better prognosis in TNBC [55,56]. Yet, their role in anti-tumor immunity is often compromised [57]. While tumor-infiltrating DCs were shown to enhance anti-tumor immune response by cross-presenting clonal neoantigens [58], their function in TNBC may be impaired by immunosuppressive TME components [59].

Dysfunction of DCs in TNBC occurs through multiple interconnected mechanisms that severely compromise antitumor immune responses. Tumor-derived exosomal factors, particularly integrin beta-2 (ITGB2), suppress TLR4 expression on DCs, leading to impaired DC maturation and reduced cytokine secretion. Additionally, tumor-associated factors promote the development of tolerogenic dendritic cells (tol-DCs) that express high levels of immunosuppressive molecules and produce IL-10 rather than IL-12, creating an immunosuppressive environment. The TME of TNBC also blocks DC differentiation at early stages through factors like putrescine, while matrix metalloproteinase-9 (MMP-9) expression further impairs DC antigen presentation capabilities by reducing MHC class I/II molecule expression. The impaired DCs fail to properly activate CD8+ and CD4+ T-cells, which results in decreased T-cell infiltration, reduced cytolytic activity, and compromised neoantigen recognition, ultimately potentiating tumor immune evasion [60,61].

### 2.4. Immunosuppressive Cell Populations

Tregs and M2-polarized macrophages secrete inhibitory cytokines (e.g., IL-10, TGF-β) and deplete metabolites like tryptophan via indoleamine 2,3-dioxygenase (IDO) [18,62,63], creating an immunosuppressive environment where even immunogenic (“hot”) tumors evade destruction [28,62]. For instance, Tregs in TNBC have been shown to directly suppress neoantigen-specific CD8+ T-cells through CTLA-4-mediated dendritic cell inhibition and adenosine receptor signaling. Moreover, IL-10 secreted by Tregs may potentiate the transformation of macrophages to M2-polarized macrophages along with inhibition of the DCs maturation, which, in turn, prevents DC-mediated antigen presentation and hinders tumor reduction [64,65]. Another notable immunosuppressive component of TME is cancer-associated fibroblasts (CAFs) [66], which secrete ECM proteins like collagen and fibronectin, which physically block DC-tumor cell interactions, while soluble factors such as prostaglandin E2 (PGE2) inhibit DC maturation [67,68].

### 2.5. TME Modeling Strategies

The balance between immune activation and suppression in the TME in TNBC is especially vital since it ultimately determines the outcomes of neoantigen-directed therapies. For example, TNBCs with high CD8+ T-cell/Treg ratios and spatially organized “tertiary lymphoid structures” (TLS) are more likely to sustain neoantigen-specific immunity, as evidenced by prolonged responses to pembrolizumab in the KEYNOTE-086 trial [69]. Conversely, tumors where myeloid-derived suppressor cells (MDSCs) or M2 macrophages prevail exhibit muted neoantigen immunogenicity, often associated with adaptive resistance to ICIs [70].

In the last few years, TME reprogramming approaches have been developing [71]. For instance, intratumoral DC activation correlates with the STING pathway that potentiates interferon type I (IFN-I)-based TME modulation and tumor regression [72]. The strategies to enhance DC function, such as intratumoral delivery of STING agonists [73] have shown promising results in preclinical TNBC models [74], synergizing with neoantigen vaccines to amplify T-cell infiltration and tumor control. Other important examples here are CSF1R inhibition to deplete immunosuppressive macrophages [75,76] or CXCR4 antagonists to disrupt CAF-ECM barriers [77,78]. These emerging strategies aim to prevent the shift in TME into immunosuppression and recruit its components in favor of neoantigen recognition.

## 3. Neoantigen Identification and Prioritization

Neoantigens originate from the aberrations occurring on different stages of protein biosynthesis in cancer cells [23]. Genomic alterations, including somatic mutations and gene fusions, are the primary source of TSAs [79,80,81]; however, neoantigens are also generated on transcription [82,83,84] and translation/post-translation [85,86] levels. The first discovery of a tumor-specific peptide that elicited a cytotoxic T-cell response was made in a murine mastocytoma model using molecular cloning methods [87]. Since then, advances in molecular biology, particularly high-throughput sequencing technologies and computational prediction, have revolutionized our ability to identify neoantigenic sequences. This section covers the comprehensive contemporary pipeline from neoantigen discovery to clinical application, discussing both established methodologies and emerging innovations in the field, as well as the current progress and perspectives of neoantigen discovery in TNBC.

### 3.1. Neoantigen Identification: High-Throughput Sequencing Opportunities

The basis of the neoantigen discovery process is the comprehensive genomic and transcriptomic profiling of tumor and matched normal tissues [88]. High-throughput sequencing (HTS) technologies have massively evolved in recent decades, along with increased affordability. To date, whole exome sequencing (WES) is the routine method of choice for tumor profiling [89], allowing the identification of somatic mutations in protein-coding regions with high sensitivity [90,91]. Whole genome sequencing (WGS) provides comprehensive information on the total tumor DNA sequence [92], but it is less affordable and requires substantial computer facilities for data analysis and storage; thus, the usage of WGS in neoantigen search is limited [93]. Contemporary WES protocols typically consider sequencing depths of 150–250× for tumor samples and 60–100× for matched normal samples as the best practice, balancing detection sensitivity with cost-effectiveness [94,95].

Genomic data can be complemented with transcriptomic data derived from RNA sequencing (RNA-seq) to evaluate the expression of mutations at the RNA level [96]. Matching RNA-seq data with the tumor DNA profile helps detect the tumor-specific splice variants [97] and gene fusions [98], and quantitative expression data are used for the prioritization of candidate neoantigens, revealing post-transcriptional modifications that may influence immunogenicity [99].

Recent studies show that integration of RNA expression data and WES results substantially improves the neoantigen recognition, leading to an increase in the number of candidate neoantigens and helping reveal the RNA signatures that possess higher immunogenic properties [100,101,102].

### 3.2. Neoantigen Prediction and Prioritization

The data obtained from DNA and RNA sequencing are then processed by specialized computational pipelines to predict and prioritize candidate neoantigens. Prioritization of neoantigens is essential because only a small fraction of TSAs provide immunogenic epitopes capable of eliciting robust T-cell responses [103,104,105]. In a nutshell, computational algorithms for neoantigen selection are based on the prediction of the strongest MHC-binding peptides (8–11-mer for MHC class I, 13–25-mer for MHC class II) [106,107] along with HLA typing and filtering based on such parameters as sequencing quality, allele fraction, etc. [108]. The first software instruments for TSA prediction emerged about a decade ago [108,109,110]. However, these tools were not able to provide the full evaluation of all types of potentially neoantigenic sequences, for instance, gene fusion-derived ones. In 2020, Hundal et al. presented the comprehensive toolkit for neoantigen characterization—pVACtools [111]. The pVACtools software provides mutation detection, HLA typing, and epitope prediction within an integrated workflow. Its modular architecture allows flexible analysis while maintaining reproducibility across studies, thus making pVACtools a convenient and widely used methodology for neoantigen investigation. The newest application, pVACview [112], enables extensive additional analysis of predicted neoepitopes, offering the full-cycle processing from raw HTS data to the preclinical and clinical trials. In the last few years, alternative “end-to-end” pipelines (such as NeoDisc [113], NeoHunter [114]) have been developed, recruiting artificial intelligence and deep learning with the goal of improving the established methods. However, the false-positive and false-negative rates of practically all existing algorithms remain high, especially for less common HLA alleles, while the ways to improve them, such as advanced machine learning and multi-omics data integration, require substantial resources, which is why their utilization often remains complicated [115].

#### 3.2.1. Neoantigen Prediction Tools

At the beginning of the process of in silico neoantigen prediction, the Variant Call Format (VCF) files are annotated and processed with MHC prediction tools such as NetMHCpan [116], NetMHCIIpan [117], MHCflurry [118], MHCnuggets [119], etc. The latest versions of such tools, like NetMHCpan-4.1, incorporate training on mass spectrometry-derived immunopeptidome datasets, significantly enhancing prediction for less common HLA alleles [120]. In recent years, the newer generation tools for MHC binding prediction have emerged, such as MARIA (MHC Analyzer with Recurrent Integrated Architecture), which utilize deep learning approaches to predict immunogenicity beyond simple MHC binding, incorporating features related to T-cell receptor recognition probability [121,122].

The aforementioned approaches typically return hundreds to thousands of candidate neoantigens [123], necessitating robust prioritization strategies to select the most immunogenic targets for experimental validation and further clinical application. The standard ways to prioritize and select neoantigens are based on the evaluation of HLA binding affinity, tumor-specific expression, and T-cell receptor (TCR) recognition.

#### 3.2.2. HLA Binding Affinity

The immunogenicity of TSAs hinges on strong HLA binding affinity. Contemporary prediction algorithms classify candidates based on predicted binding strength, typically expressed as IC50 values (nM) or percentile ranks. Computational methods usually consider the peptides with predicted IC50 values < 500 nM (for MHC-I) or percentile ranks < 2% (i.e., top 2% of all predicted binders) as strong candidates with higher probability of inducing T-cell responses [124]. However, recent evidence suggests that the existing methods may be insufficient to predict immunogenicity: the vast majority of modern software tools for epitope prediction focuses on the prediction of peptide presentation and peptide-MHC binding, but none of these processes actually characterize immunogenicity, as even non-immunogenic peptides can be presented and bind to MHC. This knowledge gap is especially remarkable in MHC class II-epitope prediction: the published studies on the matter are scarce, which limits the development of neoantigen-based personalized vaccines. The recently presented FIONA (Flexible Immunogenicity Optimization Neural Network Architecture) tool demonstrates an unprecedented capacity to predict the MHC-II epitopes’ immunogenicity through deep learning [125].

#### 3.2.3. Tumor-Specific Expression

Transcriptomic data enable expression-based filtering of candidate neoantigens, which means the selection of peptides derived from actually transcribed mutations. Expression from the RNA-seq data is usually measured in transcripts per million (TPM). Studies have shown that applying an mRNA expression threshold of >0.5–2 TPM significantly enriches clinically relevant neoantigens [126,127,128]. Recent studies demonstrate that robust neoantigens are expressed at no less than 10–15 TPM [129,130].

#### 3.2.4. T-Cell Receptor Repertoire Analysis

Each neoantigen bound to a specific MHC molecule can be recognized by a variety of TCRs, forming neoantigen-specific TCR repertoires. TCR sequencing and repertoire analysis enable clonal expansion tracking of certain epitope-specific T-cells, thus revealing the most immunogenic TSAs associated with a strong immune response [89]. Emerging techniques such as MANAFEST (Mutation-Associated Neoantigen Functional Expansion of Specific T-cells) allow multiplexed screening of neoantigen-specific T-cell responses [131]. Single-cell RNA sequencing paired with TCR sequencing [132] further makes it possible to identify expanded T-cell clones within the TME, highlighting the naturally occurring anti-tumor immune responses [133].

### 3.3. The Landscape of Neoantigens in TNBC—State of the Art

The first report of immunogenic neoantigens in TNBC was provided by Zhang et al. [134] in 2017 based on a WES and RNA-seq combined approach to the patient-derived xenografts (PDXs). That study revealed the abundance of somatic mutations in PDXs, however, resulting in few actual (triggering detectable T-cell responses) neoantigens per patient. Further works through improved neoantigen prediction demonstrated that the occurrence of putative neoantigens in TNBC samples was statistically higher than in other BC subtypes [135,136] and that the higher TMB (directly corresponds to higher neoantigen load) typically correlates with higher immune infiltration levels and pCR rates after ICI therapy [137,138]. Of note, the median TMB in TNBC is low when compared to other cancer types [138,139], which restricts the applicability of this parameter for prognosing ICI therapy outcomes to a small proportion of patients—about 10% [140,141]. Nevertheless, recent studies show that neoantigen production in TNBC is sufficient to elicit vaccine-driven immune response, which makes TNBC neoantigen investigation a priority for the further development of immunotherapy [142].

The neoantigens in TNBC mainly arise from genomic alterations, of which single-nucleotide variants (SNVs) in driver genes like *TP53* and *PIK3CA* are the most common sources [143,144]. The mutational rate estimates for *TP53* reach about 70–80% across the studies [145,146], which is significantly higher than in other BCs [147]. Somatic mutations in *PIK3CA* occur in 20–40% of TNBC cases [147,148], with *PIK3CA* and *TP53* alterations being mutually exclusive, according to Zhou et al. Numerous studies also mention other frequently mutated genes in TNBC such as *PTEN*, *AKT1*, *BRCA1*, *BRCA2*, *ATM*, *KRAS*, etc. [92,149,150]. Saravia et al. established the pool of seven genes connected mainly to the ECM maintenance and cell signaling (namely, *TTN*, *HMCN1*, *RELN*, *PKHD1L1*, *DMD*, *FRAS1*, and *RYR3*), which are enriched with mutations up to 30% specifically in metastatic TNBC (mTNBC). These results reflect the TME remodeling in cancer progression and show substantial differences in the putative neoantigen landscapes of primary and metastatic tumors [151]. On the contrary, the mutational rate for mismatch repair (MMR) genes in TNBC is only about 3%, which is significantly higher than in other BC subtypes but low compared to other cancers; nevertheless, Yan et al. demonstrated that the MMR mutations contribute strongly to the neoantigen load estimates in BCs [152]. Interestingly, indel mutations, which occur more rarely but provide higher immunogenicity, are relatively enriched in BC [153] and correlate with *BRCA1* mutational rate in TNBC samples [154]. Postgenomic sources of neoantigens, such as aberrant RNA splicing or post-translational modifications (PTMs), remain understudied in TNBC but may contribute to additional neoepitopes [155,156].

The neoantigen repertoire, i.e., the immunologically actionable fraction of TNBC’s mutational spectrum, is dominated by individual (“private”) neoantigens, whereas shared (“public”) neoantigens derived from recurrent variants occur rarely. A study by Di Cosimo et al. revealed that *TP53* is practically the only gene with a significant rate of recurrent mutations in TNBC, while other genes, such as *PIK3CA*, *KRAS*, and some others, mutated in two patients at most [157]. Moreover, Ruangapirom et al. demonstrated that, in *BRCA1*-mutated BC (more than half of which exhibit triple-negative phenotype), the spectra of recurrent somatic alterations vary across different cohorts, with *TP53* R175H the only unanimously frequently repeated variant [158]. Few studies report some other shared immunogenic variants in TNBC such as *PIK3CA* H1047R, N345K and E542K, *TP53* Y220C and R196* [158,159]. However, the vast majority of TNBC neoantigens are unique for each patient [142,144,157], and, additionally, even the immunogenicity of known shared mutations may vary across patients; for instance, Zhang et al. observed *TP53*-derived candidate neoantigens in 6 of 18 TNBC patients, but the immunogenicity of a *TP53* alteration was experimentally confirmed in only one patient [144]. The available data on the neoantigen landscape of TNBC are summarized in Table 1.

While the lack of “public” neoantigens is a hurdle for the timeliness and affordability of immunotherapeutic approaches, recent studies show that “private” neoantigens are substantially more immunogenic and preferred by the tumor-specific TCRs [160,161], which offers even more efficiency for personalized therapies.

## 4. Adoptive Cell Therapies

Adoptive cell therapy (ACT) utilizes the patient’s own lymphocytes, activated and directed against specific antigens. The studies by the pioneer team of Rosenberg et al. in the 1980s established the principles and the rationale for ACT, resulting in the first successful demonstration of the efficacy of the autologous TIL therapy in up to 50–70% of patients with metastatic melanoma in 1988 [162]. Nowadays, ACT approaches are used in various fields, including the treatment of autoimmune diseases and transplantology [163]; however, the main area of application remains cancer treatment [164]. Based on the mechanism of action, the ACT methods can be widely divided into the following principal modalities: using chimeric antigen receptor (CAR)-engineered T-cells (CAR-T cells, CAR-Ts); using T-cells with engineered TCR (TCR-T cells); and tumor-infiltrating lymphocyte (TIL) therapy recruiting and expanding the autologous TILs from the patient’s tumor samples. The technology of CAR engineering gained recognizable success and gave rise to a series of alternative CAR-based ACT approaches using alternative cell populations; of those, particularly interesting are the CAR-engineered natural killer cells (CAR-NK cells, CAR-NKs) [165]. In recent years, ACT-based therapeutic approaches to TNBC have been evolving, capitalizing on its aforementioned properties such as high TMB and abundant TIL infiltration [166].

### 4.1. Tumor-Infiltrating Lymphocyte (TIL) Therapy

The established method of autologous TIL therapy involves isolation of patient-specific TILs from tumor samples obtained during surgery and their expansion through the rapid expansion protocol (REP) [167,168]. In order to amplify the proportion of specific tumor-reacting cells in the dissociated tumor specimens, immunochemical cell sorting methods are utilized to select functional subpopulations of TILs, notably those expressing programmed cell death protein-1 (PD-1) [169]. The TIL-based approaches have shown robust clinical benefit in the treatment of melanoma, resulting in the recent FDA approval of TIL-based medication lifileucel for the treatment of inoperable or metastatic melanoma [170], which is a major milestone in this field; however, the reported efficacy in other types of solid tumors is variable [171,172].

There are a few studies regarding TIL efficacy in BC and in TNBC, specifically. Zacharakis et al. [173] presented a groundbreaking case in which a patient with HR+/HER2–metastatic BC achieved complete and durable tumor regression following treatment with TILs selected for reactivity against neoantigens. The researchers performed whole-exome and transcriptome sequencing of the patient’s tumor to identify somatic mutations, then screened the TIL population for T-cells that recognized neoantigens derived from four altered proteins: SLC3A2, KIAA0368, CADPS2, and CTSB. The mutation-reactive TILs were then expanded ex vivo and infused back into the patient, leading to complete remission. Ultra-deep sequencing was used to track the presence of neoantigen-reactive expanded T-cell clones. In subsequent publications on the same clinical trial, the authors reported tumor regression in 3 of 6 BC patients treated with neoantigen-specific TILs [174]. Chun et al. performed a preclinical study of marrow-infiltrating lymphocytes (MILs), which are tumor antigen-specific T-cells isolated from BC patients’ bone marrow, using blood and tumor samples from a group of patients with metastatic and high-risk early BCs, including three patients with TNBC [175]. In that study, MIL treatment resulted in enhanced cytokine production, which authors consider promising for future clinical trials of MILs in combination with ICIs. However, despite these inspiring early results, the recently completed NCT04111510 phase II clinical trial of autologous TILs as single therapy for mTNBC did not show clinical benefit, resulting in an ORR of 16.7% (1/6 patients), a median PFS of 46.5 days, and median overall survival of 156.5 days [176]. The latter is consistent with the previous contradictory results of clinical trials of TIL therapies for solid tumors other than melanoma. Nevertheless, the potential of TIL therapy across solid tumors, including BC-specific applications, continues to be explored: a number of clinical trials regarding autologous TILs for solid cancer therapy are active at the moment [177,178]. Regarding neoantigen-focused approaches and/or TNBC-specific applications of the TIL-based ACT, two ongoing clinical trials are of particular interest. The NEXTGEN-TIL trial is focused on the recognition and efficient selection of neoantigen-reactive TILs in order to make the most of neoantigen potential and produce a highly immunogenic preparation for the therapy of solid tumors [179], and the TILS001 trial is concentrated on the specific selection of PD1-positive TILs for the therapy of mTNBC, representing a promising advancement in TIL therapies. Also of note is the recent work by Coman et al. proposing ultrasound-guided core needle biopsy as an alternative method of TNBC specimen harvesting for the following isolation and further processing of immunotherapeutic TILs. This minimally invasive method of sampling is much less traumatic compared to traditional resection and opens the way to immunotherapies for patients with inoperable or multiple tumors [180].

### 4.2. CAR-T Therapy

CAR-T therapy is the most renowned and developed ACT approach. CAR-Ts are genetically engineered autologous cells that express a synthetic receptor (CAR) recognizing a tumor cell surface protein. A CAR construct is composed of an extracellular antigen-binding domain derived from a monoclonal antibody fragment and intracellular signaling modules such as CD3ζ and costimulatory domains (for instance, CD28 or 4-1BB). Upon reinfusion to the patient, CAR-Ts traffic to tumor sites and target the tumor cells bypassing the MHC antigen presentation. The CAR binding to the tumor antigen via the specific single-chain variable fragment (scFv) launches phosphorylation of immunoreceptor tyrosine-based activation motifs in CD3ζ, recruiting ZAP-70 and triggering downstream signaling cascades (PLCγ1, MAPK, NF-κB) that drive T-cell proliferation, cytokine secretion, and cytotoxic granule release. The co-stimulatory domains amplify these signals by enhancing PI3K/Akt and NF-κB pathways, thereby sustaining T-cell survival and promoting robust, MHC-independent cytotoxicity [181].

In recent years, a number of CAR-T cell preparations have been approved by the FDA for the treatment of hematological malignancies [182]. However, the major hurdle limiting the wide use of CAR-Ts is the targeting of nonspecific molecules, which leads to serious cytotoxicity [183]. Moreover, in solid tumors, the efficacy of CAR-T therapies is challenged by the immunosuppressive TME components. Consequently, the ways to enhance the survival and performance of CAR-T cells are emerging, such as additional engineering of CARs to improve their persistence, targeting multiple tumor antigens simultaneously, and combining with ICIs or other targeted therapies [184,185]. Several strategies for the CAR-T therapy of BC are currently under research, though there are not many studies regarding the CAR-Ts specifically for TNBC, and these studies are mainly focused on targeting TAAs rather than neoantigens.

For instance, epidermal growth factor receptor (EGFR)-targeting CAR-Ts have repeatedly shown success in suppressing TNBC both in vitro and in vivo [186,187], and the efficacy increased when combined with radiotherapy [187]. A recent study by Subham et al. also demonstrates hopeful perspectives of the use of EGFR-CAR-Ts to specifically target the brain metastases of TNBC [188], which highlights EGFR as a robust target for CAR-T-based approaches to mTNBC therapy. Other prospective TAA targets in TNBC are carcinoembryonic antigen (CEA) [189] and TEM8/ANTXR1 integrin-like protein [190], which have shown promising results in recent preclinical studies.

Clinical trials of CAR-Ts for TNBC are few, and the published output data is even less. For example, the NCT03060356 trial of c-Met-specific CAR-Ts for advanced melanoma or breast cancer [191] was terminated in 2020 with no results published, despite the previous inspiring results on the safety and efficacy of intratumoral delivery of c-Met-CAR-Ts [192]. Similarly, although preclinical research demonstrated the efficacy of receptor tyrosine kinase-like orphan receptor 1 (ROR1)-targeting CAR-Ts against TNBC [193], both clinical trials initiated to date were also terminated [194,195].

As the CAR-Ts’ persistence and activity in TNBC are significantly hindered by the immunosuppressive TME, the strategies to boost immune recognition and cytotoxicity of CAR-Ts are being developed. For instance, a novel bispecific CAR-T engaging both mesothelin and NKG2D ligands shows improved preclinical efficacy against TNBC when compared to mesothelin-only specific CAR-Ts [196]. Another innovative approach in this field is the so-called “multi-armored” allogeneic MUC1-targeting CAR-Ts, which carry numerous genetically engineered enhancers of antigen recognition and immune response, such as knocked-out PD1 and TGFBR2. The multi-armored CAR-Ts provided significant regression of TNBC when delivered intratumorally [197] and reached the phase I/II clinical trial, which is now completed; however, the results have not been published yet [198].

### 4.3. CAR-NK Therapy

CAR-NK cells continue the concept of CAR-Ts but demonstrate more robust and specific tumor targeting with less off-target toxicity due to the expression of NKG2D, CD266, and other NK cell surface markers [199]. Natural killers are innate lymphoid cells capable of rapid, MHC-independent eradication of tumor cells. Antigen recognition by CAR-NK cells occurs via the CAR scFv, bypassing the MHC presentation. The CAR engagement triggers NK cell activation, leading to degranulation and release of perforin and granzymes, which induce apoptosis in the targeted tumor cell. Activated CAR-NKs also secrete cytokines (e.g., IFN-γ, TNF-α) that recruit and activate additional immune effectors. They also retain CD16 expression, which enables antibody-dependent cellular cytotoxicity (ADCC) against opsonized tumor cells. Compared to CAR-Ts, CAR-NKs have a lower risk of graft-versus-host disease and can be used in an ‘off-the-shelf’ format [200]. The pioneering preclinical study of the CAR-NK approach to TNBC in 2020 involved tissue factor-targeting cells and showed efficacy and safety on murine models [201]. The latest advances in this field are two studies published in 2024.

Eitler et al. [202] revealed that CAR-NK cells can overcome tumor immune escape from monoclonal ADCC, which is carried out through the downregulation of the adhesion molecule ICAM-1. The study showed that the low expression of ICAM-1 in BC cells creates resistance to trastuzumab-triggered ADCC, whereas genetically engineered CAR-NKs remain effective regardless of ICAM-1 expression levels. This finding highlights the high potential of CAR-NK cells as immunotherapeutic agents and opens up the perspectives of antibody–CAR-NK combinational approaches.

Liu et al. [203] genetically engineered CAR-NKs targeting HER1-overexpressing TNBC with catalase, so that the cells, which they called HER1-CAR-CAT-NK, could eliminate excessive reactive oxygen species produced by the immunosuppressive TME, thus tolerating and overcoming the unfavorable intratumoral conditions such as high oxidative stress and hypoxia. The authors also used the injectable alginate hydrogel to enhance the sustainability of HER1-CAR-CAT-NK within the tumor foci. The resulting genetically enhanced HER1-CAR-CAT-NK exerted prominent therapeutic effects, leading to the robust suppression of both primary and distant tumor foci, thus demonstrating promise in the postoperative treatment and relapse prevention.

### 4.4. TCR-T Cell Therapy

The TCR-T approach is based on natural T-cell receptors with minimal modifications, allowing specific recognition of tumor antigens presented in complex with specific HLA molecules. Unlike CAR-Ts, TCR-T cells recognize MHC-presented antigens in a conventional way through their TCR, initiating a cascade of immune response reactions, including cytotoxic pathways, elevated cytokine production, and immune activation, which hampers tumor progression [204]. Several preclinical studies evaluated this technology for TNBC treatment. In one study, it was shown that ROPN1 is a TNBC-selective antigen (expressed in ~90% of tumors and absent in normal tissues), and a TCR specific for its FLYTYIAKV epitope demonstrated high specificity, potent cytotoxicity in 3D tumoroids and in vivo models, outperforming standard treatments. The GMP-grade T-cell product expressing this TCR passed rigorous preclinical safety and efficacy testing, highlighting its promise as a therapeutic candidate [205]. Another study identified zona pellucida glycoprotein 4 (ZP4) as a novel and highly specific tumor-associated antigen for TNBC through integrative analysis of transcriptomic and proteomic datasets. The ZP4 expression was confirmed in TNBC tissues but was absent in healthy adult tissues, highlighting its potential as a safe and effective target for future CAR-T cell therapy development in TNBC [206].

## 5. Neoantigen-Based Vaccine Platforms

Neoantigen vaccines are an emerging strategy in TNBC immunotherapy, aimed at inducing patient-specific T-cell responses against tumor-exclusive mutated epitopes. Several technological platforms are currently advancing toward clinical application, each with distinct advantages and challenges. Peptide-based vaccines are among the most established, offering ease of synthesis and favorable safety, though often requiring adjuvants [207]. Nucleic acid vaccines, including DNA and mRNA formats, allow rapid personalization and expression of multiple epitopes; recently, mRNA platforms are gaining popularity due to their success in infectious disease immunization [208,209]. Dendritic cell-based vaccines leverage the body’s own antigen-presenting cells but are more labor-intensive and costly [210]. Collectively, these platforms form the foundation for personalized, neoantigen-driven immunotherapy strategies in cancer.

### 5.1. Peptide-Based Vaccines

To date, the majority of clinical trials on anti-TNBC vaccines focuses on peptide platforms, emphasizing this approach as the most popular in the field [211]. Some personalized peptide vaccine (PPV) approaches are based on the use of several preliminary characterized TAAs, selected for their immunogenicity. A phase II study (UMIN000001844) assessed PPV in 79 patients with metastatic BC, including 18 with TNBC. Each patient was administered up to four peptides, chosen from a pool of 31 TAA-derived candidates, based on their capacity to induce strong peptide-specific IgG responses. Within the TNBC subgroup, two patients exhibited objective responses—one achieving a complete response (CR) and the other a partial response (PR). The median PFS and overall survival (OS) in this group were 7.5 and 11.1 months, respectively [212]. To boost immunogenicity, investigators designed KRM-19, a multi-peptide vaccine composed of 19 peptides derived from 11 TAAs, and evaluated it in mTNBC. In a phase II trial (UMIN000014616) involving 14 heavily pretreated advanced breast cancer patients, six weekly injections of KRM-19 yielded a clinical benefit—defined as disease control—for 62.5% of the cohort that completed the full regimen, with a median overall survival of 24.4 months. The KRM-19 vaccine elicited stronger antigen-specific IgG responses compared to prior, smaller peptide vaccines [213].

More innovative and adaptable approaches based on TSA utilization are currently in earlier phases of research. The PNeoVCA (Personalized Neoantigen Peptide-Based Vaccine) clinical trial is evaluating a personalized neoantigen peptide vaccine formulated of up to 20 15–30-mer peptides in combination with pembrolizumab in patients with solid tumors. This phase I/II trial includes both advanced and early-stage TNBC and is now on the stage of recruiting [214,215]. Also recruiting are the NCT03606967 study dedicated to the evaluation of a nab-paclitaxel + durvalumab + tremelimumab + long peptide-based neoantigen vaccine combination strategy in mTNBC [216] and the ModiFY study (NCT05329532) investigating the Modi-1/Modi-1v peptide vaccine in a number of solid tumors, including TNBC. The Mody-1 vaccine previously showed good tolerance and inspiring efficacy results as monotherapy [217]. Of note, while peptide-based neoantigen vaccines bring clinical benefits for TNBC, as well as other cancer types, their limited monotherapeutic efficacy and high manufacturing costs make their wide application unlikely, at least until further clinical research results are abundant [211].

### 5.2. DNA/RNA-Based Vaccines

Neoantigen-based nucleic acid vaccines encode patient-specific tumor mutations, enabling in situ antigen production and presentation primarily through MHC class I on all nucleated cells, while professional antigen-presenting cells (APCs) can additionally present these antigens via MHC class II, thereby stimulating both cytotoxic and helper T-cell responses. mRNA cancer vaccines have gained wider adoption compared to DNA vaccines due to several advantages: they do not require nuclear entry and ensure faster and more efficient antigen expression; they have a better safety profile by eliminating a theoretical risk of genomic integration; and they are effectively delivered using lipid nanoparticles, which have proven successful in clinical applications [218,219]. Moreover, mRNA can possess intrinsic adjuvant properties by activating innate immune responses [220].

In the first-in-human phase I trial involving patients with TNBC (NCT02348320), 18 individuals with persistent disease following neoadjuvant chemotherapy received personalized intramuscular DNA vaccines encoding an average of 11 neoepitopes per patient (range 4–20), delivered via electroporation. The vaccination was well tolerated, with adverse events limited primarily to local injection site reactions. Neoantigen-specific T-cell responses were detected in 14 out of 18 patients, confirmed by both ELISpot and intracellular cytokine staining assays. At a median follow-up of 36 months, the recurrence-free survival rate was 87.5% (95% CI: 72.7–100%) [144].

In a phase 1 clinical trial evaluating the individualized RNA-lipoplex vaccine autogene cevumeran in patients with advanced solid tumors, TNBC emerged as one of the tumor types with the highest frequency of vaccine-induced immune responses. Among ten evaluable TNBC patients, 80% demonstrated ex vivo-detectable neoantigen-specific T-cell responses, indicating robust immunogenicity of the vaccine in this population. However, despite the strong immune activation, the ORR in the checkpoint inhibitor-naive TNBC expansion cohort receiving 25 µg autogene cevumeran in combination with atezolizumab (n = 21) was 0%, suggesting that immune priming by the vaccine did not translate into measurable tumor regression in this setting. At the same time, one patient from the dose-escalation cohort with PD-L1-high disease, receiving 38 µg autogene cevumeran in combination with atezolizumab, achieved a PR lasting for 9.9 months (29.9 months of follow-up) [221].

### 5.3. Dendritic Cell-Based Vaccines

The DC-based vaccine platform has been considered especially attractive for cancer immunotherapy due to the ability of DCs to elicit strong immune responses through the activation of both MHC-I and II-dependent antigen recognition [222]. Nevertheless, despite several decades of research and promising early results in a number of studies, DC-based vaccines failed to achieve success in the majority of clinical trials [223]. To date, only one DC-based cancer vaccine for castration-resistant prostate cancer—Sipuleucel-T (Provenge)—has been approved for clinical use by the FDA [224]. The most prominent aspects limiting the development of DC-based vaccines are high manufacturing costs and long, labor-intensive production workflow [225]. Regarding neoantigen-based DC approaches, the process is further hampered by the existing difficulties in TSA detection and prediction. Nevertheless, there are a few clinical trials of DC vaccines in application to TNBC known to date. Of note is the phase I trial by the National University of Colombia (NCT04879888/NCT04105582) aimed to evaluate the safety and immunogenicity of personalized autologous DC vaccines pulsed with neoantigenic peptides in TNBC patients. At the moment, this trial is announced “complete” with restored T-cell responsiveness and no confirmed clinical benefits [226].

There are also two currently active trials dedicated to DC vaccines in combination with pembrolizumab, however focused on TAAs. The phase IIa trial by Roswell Park Cancer Institute (USA), now recruiting, investigates the efficacy of DC + pembrolizumab combination for the treatment of TNBC and HER2-positive BC brain metastases [227]. The DecipHER phase I trial focuses on early TNBC and BC with low ER expression [228]. All the above shows persisting active interest in DCs as immunotherapeutic agents, despite numerous disappointing results.

Table 2 summarizes the major results of clinical studies of neoantigen-driven immunotherapeutic platforms for TNBC.

### 5.4. Oncolytic Viruses

Oncolytic viruses offer a dual therapeutic mechanism that addresses key challenges in cancer immunotherapy. First, they selectively infect and lyse tumor cells, converting immunologically “cold” tumors into “hot” ones by exposing previously hidden neoantigens to immune surveillance [229,230]. Second, they can be genetically engineered to express additional tumor-specific peptides, thus functioning as in situ cancer vaccines [231,232]. The tumor selectivity of viruses can be boosted through genetic modifications that attenuate their replication in normal cells while preserving their ability to replicate in cancer cells [233,234]. Tumor-selective oncolytic vaccinia virus can be further modified to encode selected neoantigens derived from patient-specific mutations, additionally enhancing targeted immune responses [235]. However, while theoretically possible, this approach faces significant technical challenges. Wang et al. [236] described how oncolytic viruses can be engineered to co-express neoantigens and neoantigen-binding MHC molecules; however, this work represents cutting-edge research rather than a practically applicable approach. More realistic neoantigen-based strategies are based on delivering neoantigens alongside oncolytic virus therapy rather than inducing the direct expression of patient-specific neoantigens by the virus. Speaking of TNBC, Baleeiro et al. developed a “personalized viro-immunotherapy platform” that combined tumor-selective oncolytic vaccinia virus with separately delivered neoantigen peptides and provided promising results in a preclinical model of TNBC, eliciting tumor-specific immune response and changing the TME to attract and maintain mature cross-presenting CD8α + DCs and effector T-cells, which resulted in tumor growth inhibition and improved survival [237]. Despite the promising results across various solid tumors, the efficacy of oncolytic viruses as monotherapy may be significantly hampered by the immunosuppressive properties of TME; Cambien et al. observed in a canine TNBC model the 28-fold repression of vaccinia viral replication selectively in cancer cells, which they found to be mediated by the *DDIT4* gene expression [238]. Other notable barriers are the organism’s immune response to the virus and intratumoral delivery issues, which is why the main future directions of vaccinia-based approaches in TNBC seem to be the development of different combination strategies and the improvement of delivery options [239,240,241].

The summary of the aforementioned neoantigen-based immunotherapeutic approaches, its effects, advantages, and disadvantages is presented in Table 3.

## 6. Conclusions and Perspectives

Neoantigen-driven immunotherapeutic approaches represent a paradigm shift in TNBC treatment, embodying the concept of precision medicine. While current approaches demonstrate proof-of-concept for personalized cancer vaccines and adoptive cell therapies, several challenges must be addressed to realize their full clinical potential. Improving neoantigen prediction accuracy, optimization of manufacturing costs, and development of combination strategies will be crucial for translation of neoantigen-based methods to routine clinical practice. The unique immune properties of TNBC, characterized by high mutational burden and abundant immune infiltration, provide both opportunities and challenges for neoantigen-driven approaches. We consider that integrating neoantigen-based solutions with existing therapeutic strategies, optimizing treatment timing, and streamlining clinical-grade vaccine development workflows will be essential to ensure broad patient access to neoantigen-based personalized immunotherapies in the future.

## Figures and Tables

**Table 1:** Neoantigen sources in TNBC and their immunogenic potential.

Group	Gene(s)/Mutation(s)	Frequency/Features	Neoantigen/Immunogenic Potential	References
I. Driver mutations	*TP53*	~70–80% of TNBC; higher than in other BC subtypes	Frequent candidate neoantigens; recurrent variants: R175H, Y220C, R196*; immunogenicity confirmed only in a minority of cases	[143,144,145,146,147,157,158,159]
	*PIK3CA*	20–40% of TNBC; mutually exclusive with *TP53*	Shared immunogenic variants: H1047R, N345K, E542K; variable immunogenicity	[147,148,149,157,158,159]
	*PTEN*, *AKT1*, *BRCA1*, *BRCA2*, *ATM*, *KRAS*	Less frequent than *TP53*/*PIK3CA* but repeatedly reported; *KRAS* recurrent in ≤2 cases	May contribute to neoantigens; especially relevant in *BRCA1*-associated TNBC	[92,149,150,157]
II. Non-driver mutations	*TTN*, *HMCN1*, *RELN*, *PKHD1L1*, *DMD*, *FRAS1*, *RYR3*	Mutated in up to 30% of metastatic TNBC; linked to ECM and signaling	Reflect TME remodeling; distinct neoantigen landscapes in metastases	[151]
	MMR genes (*MLH1*, *MSH2*, *MSH6*, *PMS2*, etc.)	~3% of TNBC; higher than in other BCs but low overall	Strong contribution to neoantigen load when mutated	[152]
	Indels	Less frequent than SNVs but more immunogenic; enriched in *BRCA1*-mutated TNBC	Broaden neoepitope diversity; high immunogenicity	[153,154]
III. Post-genomic alterations	Aberrant splicing, post-translational modifications (PTMs)	Still understudied	Potential additional source of novel neoepitopes	[155,156]

**Table 2:** Summary of clinical trials dedicated to neoantigen-based methods of TNBC immunotherapy.

Modality	Trial/ID	Phase	Design/Combo	Neoantigen Strategy	Key Outcomes (TNBC)	Refs.
DNA neoantigen vaccine	NCT02348320	I	Personalized plasmid DNA vaccine (average 11 neoantigens per patient), (intramuscular via electroporation)	Patient-specific mutated epitopes (WES/RNA-seq → prediction)	Neoantigen-specific T-cell responses in 14/18; 36-months RFS 87.5%	[144]
mRNA neoantigen vaccine + anti-PD-L1	NCT03289962 (Autogene cevumeran)	I a/b	Individualized RNA-lipoplex vaccine ± atezolizumab	Patient-specific mutated epitopes encoded in RNA	PR reported in 1 TNBC pt; robust neoantigen T-cell responses	[221]
Peptide neoantigen vaccine + anti-PD-1	NCT05269381 (PNeoVCA)	I	Up to 20 long peptides per patient + pembrolizumab	Patient-specific long peptides	No TNBC-specific outcomes posted yet	[214,215]
Peptide neoantigen vaccine + ICI + chemo	NCT03606967	I	Personalized long-peptide vaccine + durvalumab + tremelimumab + nab-paclitaxel	Patient-specific long peptides	Not yet reported	[216]
Autologous DC vaccine pulsed with neoantigen peptides	NCT04105582/NCT04879888	I	Patient-specific neoantigenic peptide-pulsed DCs	Ex vivo–loaded DCs with predicted patient mutations	Restored T-cell responsiveness	[226]
Neoantigen-reactive TIL therapy	NCT01174121	II (basket)	Selection/expansion of mutation-reactive TILs + lymphodepletion + IL-2	Ex vivo selection of TIL clones reactive to patient-specific mutated epitopes	Across BC, tumor regression in 3/6 in one series; TNBC-specific outcomes not isolated	[173,174,175]

**Table 3:** Summary of adoptive cell therapies and neoantigen-based platforms in TNBC.

Immuno-Therapy Approach	Subtype	Advantages	Disadvantages	Therapeutic Effects	Mechanism
Adoptive Cell Therapies (ACT)	TIL Therapy	Personalized, uses patient’s own immune cells; proven efficacy in melanoma; potential in other solid tumors	Variable efficacy outside melanoma; labor-intensive; limited by tumor accessibility	Tumor regression in select cases; ongoing trials in BC/TNBC	Expansion of tumor-reactive TILs ex vivo → reinfusion → targeted killing of tumor cells
	CAR-T Therapy	Strong, MHC-independent cytotoxicity; FDA-approved for hematologic cancers; flexible antigen targeting	Severe toxicity risks; limited efficacy in solid tumors due to immunosuppressive TME	Promising in preclinical TNBC models; some early clinical attempts	Engineered T-cells with synthetic receptors directly recognizing tumor antigens
	CAR-NK Therapy	“Off-the-shelf” potential; lower toxicity risk; combine innate and engineered immunity	Early stage of development; durability and persistence challenges	Preclinical efficacy in TNBC; promising safety profile	Engineered NK cells with CARs → direct killing, cytokine release, ADCC
	TCR-T Therapy	High specificity to intracellular tumor antigens; broad target repertoire beyond surface proteins	HLA-restricted; risk of off-target reactivity; complex personalization	Potent preclinical activity in TNBC; no mature clinical data yet	TCR-engineered T-cells recognize tumor antigens presented by MHC molecules
Neoantigen-Based Vaccines	Peptide-Based Vaccines	Simple design; established platform; relatively safe	Limited efficacy as monotherapy; high cost for personalization	Induces antigen-specific immune responses; modest clinical benefit in TNBC	Synthetic peptides stimulate T-cell activation, often with adjuvants
	DNA/RNA Vaccines	Rapidly customizable; can encode multiple antigens; strong immunogenicity	Variable clinical efficacy; manufacturing/logistics issues	Induce neoantigen-specific T-cell responses; some early success in TNBC	Delivery of nucleic acids → in situ antigen production → T-cell activation
	Dendritic Cell Vaccines	Potent immune activation via APCs; versatile	Labor- and cost-intensive; mixed clinical results	Ongoing TNBC trials; limited success so far	Ex vivo loaded DCs present tumor antigens → activate T-cell responses
Oncolytic Immunotherapies	Oncolytic Viruses	Dual effect: tumor lysis + in situ vaccination; can be engineered to carry antigens	Delivery barriers; immune clearance of virus; variable efficacy	Preclinical TNBC studies show immune activation and tumor inhibition	Viral infection of tumor cells → lysis + release of neoantigens → immune priming

## Data Availability

No new data were created or analyzed in this study. Data sharing is not applicable to this article.
